# Cerebral Surgery

**Published:** 1895-07-06

**Authors:** 


### Progress in Surgery.

### CEREBRAL SURGERY.

**Cerebral Compression.**—Professor Victor Horsley[1] discusses the mode of death in cerebral compression, and its prevention. He believes that cases of cerebral hæmorrhage, cerebral tumour, and of depressed fracture, as well as cases of sudden and violent concussion, especially when applied in the occipital region, die from failure of respiration, and not from cardiac failure. From experiments made by Professor Victor Horsley and Mr. Walter Spencer it appears that increased intra-cranial pressure causes slowing of the respiratory movements, and later their arrest; whereas after release and re-establishment of the pressure had been carried out once or twice the respiration became periodic, and exhibited the Cheyne-Stokes character. These experiments were confirmed by Dr. Leonard Hill, who believes that they are wholly due to simple anæmia of the respiratory centre. Respiratory failure, Professor Horsley considers the common end of practically all cases of pathological intra-cranial tension, especially when the development of the disease is extremely chronic. Several cases are mentioned in which, during the operation for cerebral tumour or abscess, the patient's breathing ceased, but artificial respiration was employed until the skull had been opened and the intra-cranial tension relieved, when spontaneous breathing recommenced. During some experiments on monkeys, Horsley discovered that irrigation of the head with very hot perchloride lotion had a very beneficial effect in removing the shock of the operative procedure, and in restoring the activity of the respiratory centre; and he has, since conducting these experiments, always made use of this irrigation in cerebral operations. He raises the question whether the ice-bag is the proper treatment for cases of cerebral injury, and expresses considerable doubt as to its hæmostatic effects on the intra-cranial contents. Cases of sudden death from a blow on the head, such as may occur from a cricket ball, especially when striking the occipito-temporal region, are, Horsley believes, deaths from failure of the respiratory centre, and can, he considers, be proved to be such by experiment, with unfailing accuracy. That such is the mode of death in bullet wounds of the cerebral hemispheres Horsley and Kramer[2] have lately shown. All such cases should be treated directly by the performance of artificial respiration, and heat should be applied to the head, preferably by irrigation. In cases of failure of respiration from increased intra-cranial pressure after artificial respiration has been started, the skull should be opened at once to relieve the pressure.

Mr. Leonard Hill has lately conducted some experiments on increased intra-cranial pressure and absorption of fluid within the skull.[3] He finds that the pathway of absorption of serum and normal saline solution from the intra-cranial cavity is by the blood vessels of the meninges, and not the lymphatics The brain was found to transmit increased pressure equally in all directions when pressed upon at any one point, and thus Bergman's assertion that the brain must be regarded as a bag of fluid in its physical relations proved. The intra-cranial pressure is always greater when the head is low. The intra-cranial pressure and the blood pressure is always the same within the brain. The cerebro-spinal fluid is re-absorbed as soon as the intra-cranial pressure rises above the normal. When the intra-cranial pressure is increased by the introduction of a foreign body within the cranium, the brain is so pressed down into the foramen magnum, which it blocks as a valve, that the important centres in the lower part of the medulla are protected from the increased intra-cranial pressure, as the pressure in the spinal canal remains unaffected. The posterior arch of the atlas was removed, and the pressure within the spinal canal estimated in that region.

**Injuries of the Head.**—Mr. Bryant[4] considers that all cases of so-called concussion are invariably cases of cerebral injury of a visible kind—that either bruising or laceration to some extent has taken place; and he mentions numerous cases from Guy's Hospital records to show that some definite lesion is always found after death in such cases. Such was the opinion of Sir Prescott Hewett, Mr. Hilton, and Mr. Le Gros Clarke.

Mr. Butlin records some interesting cases of obscure head injury.[5] In one case a man fell and received a blow on the occipital region. Four days after the injury he developed convulsive attacks, beginning on the left side. They were very frequent, and left some paresis after them. Although there were no external signs of injury over the right motor area, Mr. Butlin trephined over the arm and face centre, but only found a minute clot under the dura mater, and beneath this, lying under the arachnoid, an excess of cerebro-spinal fluid, with a corresponding slight depression in the brain. This fluid was evacuated. There were no fits after the operation, but his mental condition remained very unsatisfactory, and recovery only very slowly took place. In another case a large abscess, secondary to necrosis of the frontal bone from injury, was missed when the brain was explored for abscess; and in a third case a patient was trephined for secondary unconsciousness 23 days after a severe injury to the head, and nothing abnormal was discovered in the brain either then or a day later at the autopsy, the cerebral symptoms being presumably due to pneumonia, from which he was suffering.

Drs. Scudder and Lund, of Boston,[6] have collected the records of 48 cases of uncomplicated intra-cranial hæmorrhage, from American, French, German, and English literature from 1886 to 1894. Jacobson[7] collected the cases reported up to 1886. The sources of hæmorrhage were the middle meningeal artery, the sinuses, the vessels of the pia mater, and lacerated brain tissue. They found that the difficulty of distinguishing hæmorrhage from any of these sources from the associated laceration and concussion of the brain was very great; and that the only very important general symptom of intra-cranial hæmorrhage was the interval of consciousness between the time of the injury or the initial concussion and the compression from hæmorrhage. But this interval of consciousness was by no means always marked. Out of 27 cases of extradural hæmorrhage this interval of consciousness was present in only 14 cases and absent in 13; and in 21 cases of subdural hæmorrhage the interval of consciousness was only present in 9 cases. Jacobson concluded that the interval of consciousness was absent in one-third of the cases. In the cases in which there is no interval the unconsciousness of concussion passes on into that of compression. As is well known, the interval may be very brief, but they record one case where symptoms did not come on for two months. Two months after a fall paraplegia followed by hemiplegia developed. Ten days later the tongue was turned to the right. The patient became comatose. A sub-dural clot was sound over the right fissure of Rolando, was removed, and he recovered. The diagnosis of a cerebral abscess was made before the operation. The author of this paper found (as Jacobson had done before) that if the pupils did not react to light the compression was so profound that after trephining, the brain usually failed to recover. The importance of dilatation of the pupil on the same side as the injury they look on as considerable. It was originally pointed out by Hutchinson, and is due to pressure on the third nerve.

[1] The Quarterly Medical Journal, July, 1894, p. 305. [2] Proceedings of the Royal Society, 1894. [3] Brit. Med. Journal, 1894, vol. ii., p. 190. [4] Clinical Journal, 1894, Oct. 31st, p. 1. [5] Medical Week, Jan. 11th, 1995. [6] American Journal of Med. Sciences, April, 1895, p. 379.

*(To be continued.)*

